# Ketofol versus Dexmedetomidine for preventing postoperative delirium in elderly patients undergoing intestinal obstruction surgeries: a randomized controlled study

**DOI:** 10.1186/s12871-023-02378-5

**Published:** 2024-01-02

**Authors:** Shereen E. Abd Ellatif, Sherif M. S. Mowafy, Mona A. Shahin

**Affiliations:** Department of Anesthesia, Intensive Care, and Pain Management. Faculty of Medicine, Zagazig University, Zagazig, Egypt

**Keywords:** Postoperative delirium, Elderly patients, Intestinal obstruction surgeries, Dexmedetomidine, Ketofol

## Abstract

**Purpose:**

Postoperative delirium (POD) is considered the most common postoperative neurological complication in elderly patients. The aim of this study was to evaluate the efficacy of the administration of ketofol versus dexmedetomidine (DEX) for minimizing POD in elderly patients undergoing urgent exploration for intestinal obstruction.

**Methods:**

This prospective double-blinded randomized clinical trial was conducted on 120 elderly patients undergoing urgent exploration for intestinal obstruction. Patients were randomly allocated to one of the three groups: Group C (control group) patients received normal saline 0.9%, group D received dexmedetomidine, and group K received ketofol (ketamine: propofol was 1:4). The primary outcome was the incidence of POD. Secondary outcomes were incidence of emergence agitation, postoperative pain, consumption of rescue opioids, hemodynamics, and any side effects.

**Results:**

The incidence of POD was statistically significantly lower in ketofol and DEX groups than in the control group at all postoperative time recordings. Additionally, VAS scores were statistically significantly decreased in the ketofol and DEX groups compared to the control group at all time recordings except at 48 and 72 h postoperatively, where the values of the three studied groups were comparable. The occurrence of emergence agitation and high-dose opioid consumption postoperatively were found to be significant predictors for the occurrence of POD at 2 h and on the evening of the 1st postoperative day.

**Conclusion:**

The administration of ketofol provides a promising alternative option that is as effective as DEX in reducing the incidence of POD in elderly patients undergoing urgent exploration for intestinal obstruction.

**Trial registration:**

This clinical trial was approved by the Institutional Review Board (IRB) at Zagazig University (ZU-IRB# 6704// 3/03/2021) and ClinicalTrials.gov (NCT04816162, registration date 22/03/ 2021). The first research participant was enrolled on 25/03/2021).

**Supplementary Information:**

The online version contains supplementary material available at 10.1186/s12871-023-02378-5.

## Introduction

Postoperative delirium (POD) is an acute brain insult characterized by changes in a neuropsychiatric patient’s state from his or her mental function baseline [1]. POD itself is not a disease, but it is a set of symptoms, including changes in consciousness, attention, cognition, and perception. Its onset usually starts from 2 to 5 days postoperatively [2]. It is considered the most common postoperative neurological complication in elderly patients, as its incidence is approximately 20% in those > 60 years, reaching 50% in high-risk surgeries, such as hip fractures and cardiac surgery [3].

Postoperative delirium develops elderly patients due to multiple risk factors that can be separated into patient-related risk factors such as preexisting dementia, older age, functional impairment, greater comorbidities and psychopathological symptoms or operation-related risk factors such as major or emergency operations [4, 5].

Small bowel obstruction (SBO) is one of the most frequent causes of emergency surgery in the elderly population [6]. A previous study reported that approximately 10–20% of elderly patients (> 65 years) presenting with acute abdominal pain at the emergency department are diagnosed with SBO [7]. SBO is complicated by dehydration, malnutrition, electrolyte, and acid base disturbance, as well as the insertion of many catheters, such as nasogastric tubes, central venous catheters and Foley catheters, in addition to multiple drug intake due to the associated comorbidities; all these factors increase the risk of developing POD [8].

Ketamine is an N-methyl-D-aspartate antagonist that is widely used as an anesthetic drug with hypnotic and analgesic properties. Ketamine has been found to exhibit neuroprotective effects through its potential to reduce apoptosis, microthrombosis, and postoperative inflammatory markers as well as alleviate postoperative pain and opioid consumption [9, 10]. Propofol has a CNS protective effect by activating γ-aminobutyric acid receptor (GABA) receptors and suppressing the excitatory amino acid transmitter system; therefore, it protects brain cells against the oxidative stress cascade [11].

Ketofol, which is a mixture of ketamine and propofol, has gained increasing interest as an agent for procedural sedation and analgesia to produce more stable hemodynamic and respiratory profiles, as ketamine and propofol appear to counter each other’s adverse effects; the sympathomimetic effects of ketamine and dose-dependent hypotension and respiratory depression of propofol [12]. Ketofol has been used in different mixed ratios (1:1–1:10) [13–15].

Dexmedetomidine, a highly selective alpha-2 adrenoreceptor (α2) agonist, has been widely used in surgical patients and has positive sedative, anti-anxiety, and analgesic effects. DEX provides analgesia, reduces delirium-inducing medications, enhances natural sleep–wake cycles, and suppresses inflammatory processes by activating α2 receptors and stimulating the vagus nerve via a vagal- and α7 nicotinic acetylcholine receptor-dependent mechanism [16, 17]. DEX might exhibit protective effects against transient cerebral ischemia or ischemic reperfusion impairment by restraining inflammation in the brain [18]; therefore, it is considered a potential therapeutic option for the prevention and treatment of ICU delirium [19].

The objective of this current prospective randomized clinical study was to evaluate the efficacy of intraoperative and 2-h postoperative administration of DEX and ketofol for minimizing POD and postoperative pain in elderly patients scheduled for urgent exploration due to intestinal obstruction.

## Patients and methods

### Study population and design

This prospective double-blind randomized controlled study was conducted at Zagazig University Hospitals from March 2021 to February 2023. After obtaining approval from the institutional review board (The research ethics committee of the Faculty of Medicine, Zagazig University) with the reference number (ZU-IRB#6704), obtaining written informed consent from all patients and registering the study with ClinicalTrials.gov (NCT04816162), one hundred and twenty American Society of Anesthesiologists class II and III (ASA II and III) elderly patients aged ≥ 60 years old of both sexes, with BMI < 35 kg/m^2^ and able to communicate verbally scheduled for urgent exploration due to SBO under general anesthesia for at least 60 min were enrolled in this study. Patients who refused to participate in our study and patients with a history of delirium, stroke and/or transient ischemic attack, severe deafness, antipsychotics, or allergies to any drugs used in this study were excluded.

For all patients, the goal of the study was explained to clarify the advantages and possible complications and to obtain written informed consent regarding the procedure from every patient. Adequate preoperative evaluations (including detailed history, proper physical examination, and laboratory investigations such as complete blood count, random blood glucose, electrolyte, and acid‒base analysis, liver and kidney function tests and coagulation profile) were performed. For all participants in this study, mental status was assessed by using the ten-item Short Portable Mental Status Questionnaire (SPMSQ) for elderly individuals, in which 1 = correct answer and 0 = refusal. A score of < 7/10 was considered a sign of clinically significant cognitive dysfunction [20]. The patients were instructed on how to represent their pain level using the visual analogue scale (VAS), in which 0 = no pain and 10 = maximum worst pain [21]. Prior to surgery, a central venous catheter was inserted for all patients to correct intravascular volume depletion, serum electrolyte, and acid base disturbance if present.

### Sample size calculation

The frequency of postoperative delirium among patients receiving dexmedetomidine was 10%, and that among the control group was 33.3% [22], so the sample size was calculated by the open EPI program to be 120 patients (40 patients in each group) with a confidence level of 95% and power of 80%.

### Randomization

This study was conducted in a double-blind manner (the patient and the data collectors were blind to the medications used and assignment). The randomization was performed using computer-generated number tables to classify the patients into three equal groups.

**Group C** (control group): patients were infused with 0.3–0.4 mg/kg/h of 21 ml of 0.9% normal saline (1 ml of 0.9% normal saline = 9 mg of sodium chloride).

**Group D** (DEX group): patients were infused with 0.2 µg/kg/h from a solution prepared by the addition of 200 µg (2 ml) DEX to 19 ml of 0.9% normal saline.

**Group K** (ketofol group): patients were infused with 0.3–0.4 mg/kg/h ketofol, which was prepared by the addition of 50 mg ketamine (1 ml) to 200 mg propofol (20 ml) (ketamine: propofol ratio was 1:4) (i.e., rate of propofol infusion was kept at 0.3–0.4 mg/kg/h and that of ketamine was 0.125 mg/kg/h at this ratio).

Upon arrival at the operating room, standard monitors were applied to every patient, and baseline readings of heart rate (HR), mean arterial blood pressure (MAP), oxygen saturation (SPO2%), end tidal carbon dioxide (ETCO_2_), central venous pressure (CVP), and urine output (UOP) were recorded. Nasogastric tube aspiration helped us to decompress the stomach preoperatively to decrease aspiration risk.

General anesthesia was induced for all patients by using a rapid sequence induction technique where a trained assistant applied cricoid pressure; then, IV fentanyl (2 µg/kg) and propofol (1.5–2 mg/kg) were administered, and rocuronium (1 mg/kg) was given to facilitate endotracheal intubation. The lungs were ventilated using volume-controlled ventilation (tidal volume 6–8 ml/kg) to maintain ETCO_2_ of 35–40 mmHg. Anesthesia was maintained with a mixture of O_2_ and 1–1.5 MAC isoflurane, and muscle relaxation was maintained with rocuronium 0.2 mg/kg every 30 min. as well as intraoperative additional fentanyl dose (0.5 µg/kg) was given if required to maintain sufficient anesthesia depth.

Fifteen minutes after the induction of anesthesia, the attendant anesthetist who was blind to the purpose and drugs used in this study was asked to infuse the patient with one of the aseptic previously prepared foil covered 21 ml solutions by syringe pump according to the aforementioned randomization. These solutions were infused into the patient throughout the whole surgical procedure and continued for 2 h postoperatively.

Intraoperative close monitoring and recording of hemodynamics (including HR and MAP), SPO_2_% and ETCO_2_ were measured every 10 min until the end of surgery. Additionally, intraoperative monitoring of CVP, UOP and serum electrolytes were performed. Intraoperative warm intravenous fluids and blood products were used to minimize heat loss and avoid hypothermia in elderly patients. Bradycardia was defined as a decrease in HR less than 50 beats/min or a 20% decrease from the baseline value and was treated with 0.02 mg/kg atropine. Hypotension was defined as a decrease in systolic blood pressure ≥ 20% of baseline or less than 90 mmHg and was treated with warm intravenous fluid bolus and/or IV ephedrine (5 mg increments) depending on the patient’s hemodynamic state.

At the end of surgery, isoflurane was turned off, and the residual effect of rocuronium was reversed by sugammadex (2–4 mg/kg) after detecting the patient’s spontaneous respiration attempts. All patients received IV multimodal analgesia in the form of I.V. paracetamol 1 gm and 15 mg ketorolac for postoperative pain relief to be continued in the following postoperative days in the form of paracetamol (15 mg/kg 4/day) and ketorolac (0.5 mg/kg 3/day, maximum dose 60 mg/day).

Patients were transferred to the post-anesthesia care unit (PACU) extubated with standard monitors. Thereafter, patients were shifted to the ICU for finishing our study drug infusions and close monitoring of patients (HR, MAP, SPO2% measured every 15 min until the end of infusion). Emergence agitation assessed by Richmond agitation and sedation score (RASS) ranging from -5{unarousable} to + 4 {combative}, while 0 = alert and calm. If the RASS score was ≥  + 1, the patient was defined as having emergence agitation [23]. Pain severity evaluation by VAS was performed at 30 min, 1 h, 2 h, 4 h, 12 h, 24 h, 48 h and 72 h postoperatively. If VAS ≥ 3 pain relief was achieved by intravenous administration of rescue fentanyl 1 μg/kg. Postoperative rescue opioids were quantified and recorded for the postoperative 3 day.

Our primary outcome was to assess the incidence of POD by using the 2-step approach that helped patient consciousness evaluation by RASS then using the confusion assessment method for the ICU (CAM-ICU) to assess for POD [24]. CAM-ICU assessment was started 2 hours postoperatively to allow adequate patient recovery with RASS ≥ -3 and the patient is sufficiently arousable, then at the evening of the first postoperative day and twice daily for another two days in the ICU (at 11 am. and 6 pm.) to allow at least 6 hours to elapse between the two daily assessments.

In ICU tight control of intravascular volume and electrolyte balance was performed postoperatively, and wound care, antibiotic prophylaxis, and thromboembolic prophylaxis were performed when indicated. Enteral nutrition was encouraged as soon as feasible after approval of the surgeon.

### Data collection

- Patient characteristics: Age, sex, BMI, ASA physical status.
- Hemodynamics (HR and MAP) and oxygen saturation (SpO_2_) were recorded at baseline prior to surgery, after starting infusion of the study drugs, and then at 30, 40, 50 min,1 h and at the end of surgery; later, they were recorded at 15, 30, 60, 90 and 120 min postoperatively.
- The incidence of emergence agitation assessed by RASS was recorded in the PACU. If the RASS score was ≥  + 1, the patient was defined as having emergence agitation.
- Pain assessment by VAS was recorded at 30 min, 1 h, 2 h, 4 h, 12 h, 24 h, 48 h and 72 h postoperatively. Pain relief when VAS ≥ 3 was achieved by intravenous administration of rescue fentanyl 1 μg/kg. Postoperative rescue opioids were quantified and recorded for 3 days postoperatively.
- Assessment of the incidence of POD when the patient could be aroused sufficiently with RASS ≥ -3. POD was assessed using the confusion assessment method for the ICU (CAM-ICU) starting 2 h postoperatively, then at the evening of the first postoperative day and twice daily for another two days in the ICU (at 11 a.m. and 6 p.m.) to allow at least 6 h to elapse between the two daily assessments.
- Any side effects related to the study drugs as hypotension, bradycardia, hallucinations, and nightmares were noted and recorded.

### Statistical analysis

All data were collected, tabulated, and statistically analyzed using SPSS version 19. Continuous quantitative variables are expressed as the mean ± SD and median (range), and categorical qualitative variables are expressed as absolute frequencies (number) and relative frequencies (percentage). Continuous data were checked for normality by using the Shapiro Wilk test. One-way ANOVA (F test) and Kruskal‒Wallis tests were used to compare more than two groups of normally and not-normally distributed data, respectively. The least significant difference post hoc test (LSD) and Mann‒Whitney test were used to compare two groups separately. Categorical data were compared using the chi-square test and Fisher’s exact test.

The Spearman correlation test was used to detect the closeness of the association between two variables. A binary logistic regression test was used for prediction. All tests were two sided. A *P* value < 0.05 was considered statistically significant (S), a *p* value < 0.001 was considered highly statistically significant (HS), and a *p* value ≥ 0.05 was considered statistically insignificant (NS).

## Results

**Fig. 1:**
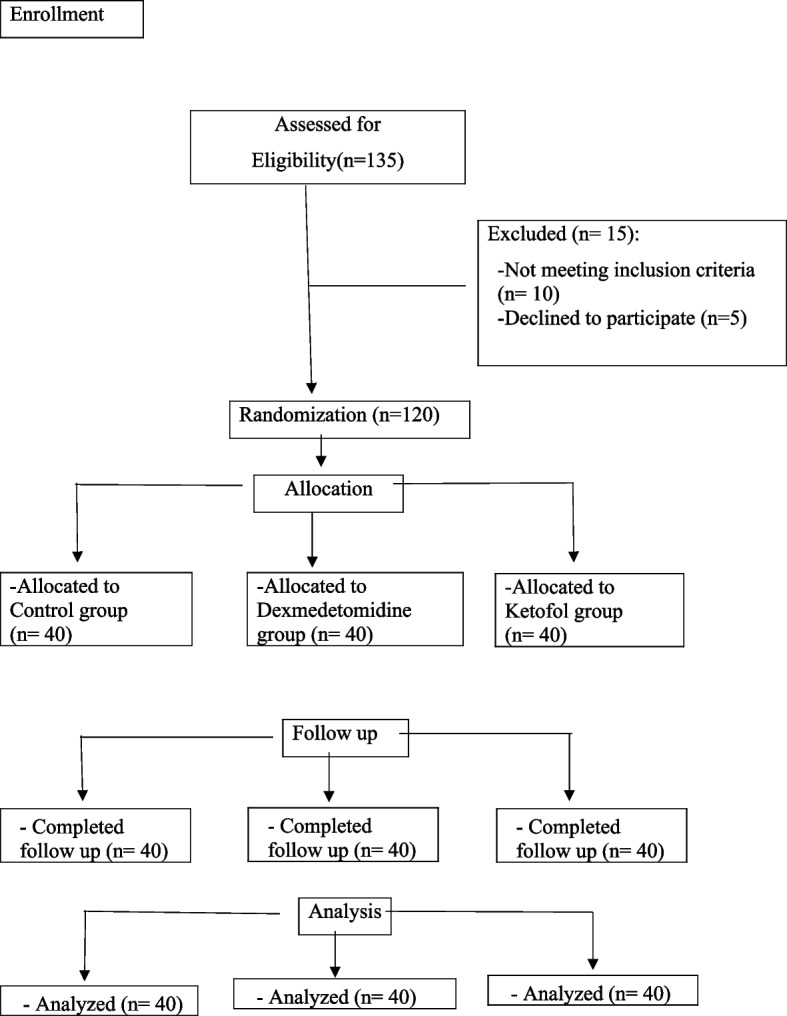
CONSORT flow diagram

A total of 135 elderly patients scheduled for urgent exploration due to intestinal obstruction under general anesthesia were evaluated for eligibility to participate in the study; 15 patients were excluded, 5 patients declined to participate, and the remaining 10 patients met one or more of the exclusion criteria. Therefore, this study included 120 elderly patients randomized into three equal groups of 40 each, as shown in the CONSORT flow diagram (Fig. 1).

There were no statistically significant differences in the three studied groups regarding age, BMI, sex, ASA status, or operation time (Table 1).

**Table 1:** Patient characteristics and operative data

Characteristics	Group C (*n* = 40)	Group D (*n* = 40)	Group K (*n* = 40)	*P*
Age (years)	71.3 ± 6.9	72.2 ± 7.7	71.6 ± 7.8	0.866^†^
BMI (kg/m2)	27.5 ± 3.1	27.6 ± 3.5	27 ± 3.6	0.731^†^
Sex Number (%)				
Female	17 (42.5%)	15 (37.5%)	17 (42.5%)	0.871^‡^
Male	23 (57.5%)	25 (62.5%)	23 (57.5%)	
ASA Number (%)				
II	25 (62.5%)	24 (60%)	25 (62.5%)	0.965^‡^
III	15 (37.5%)	16 (40%)	15 (37.5%)	
Operation time (min.)	64.8 ± 2.5	67.8 ± 2.5	68.8 ± 2.5	0.159^†^

Data are expressed as the mean ± SD, number, and percentage

*C* Control group, *D* Dexmedetomidine group, *K* Ketofol group

*n* = Total number of patients in each group

*BMI* = Body Mass Index

^†^ANOVA test

^‡^Chi-square test

*P* > 0.05 = Non significant difference

**Fig. 2:**
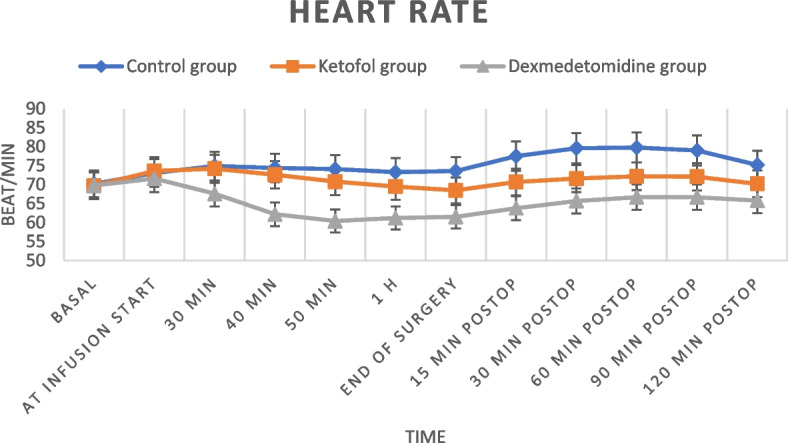
Heart rate at different times among the studied groups

**Fig. 3:**
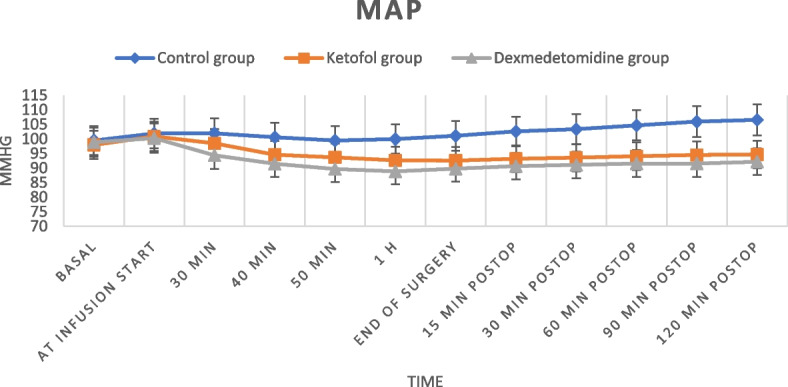
Mean arterial pressure (MAP) at different times among the studied groups

The mean values of HR and MAP were comparable at the baseline reading and at the start of drug infusion, with no significant difference between the three studied groups; later, their values in the dexmedetomidine group were statistically significantly lower than those in the control and ketofol groups (with a statistically significant difference between the last two groups as well) at all the measurement times until the end of drug infusion (Figs. 2, 3).

**Fig. 4:**
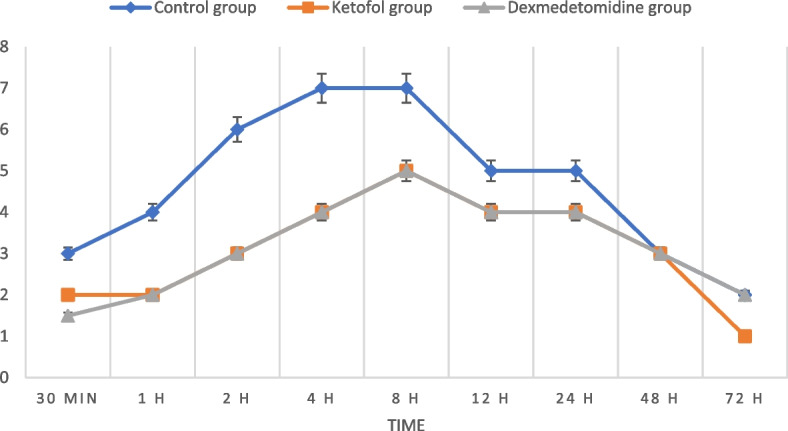
Visual analogue scale (VAS) scores at different times among the studied groups

Regarding VAS, there were highly statistically significant higher scores in the control group than in the dexmedetomidine and ketofol groups (with no significant difference between the last two groups) at all time points except at 48 and 72 h postoperatively, where the values of the three studied groups were comparable (Fig. 4).

Regarding the total consumed fentanyl in the postoperative three days, there were statistically significantly higher doses in the control group compared to the other two interventional groups (*p* < 0.05**)** (with no statistically significant difference between the last two groups themselves) (Table 2). Furthermore, there was a statistically significant increase in the incidence of emergence agitation in the control group compared to the dexmedetomidine group, with no significant difference found between the dexmedetomidine group and the ketofol group (Table 2). Throughout the entire study, there was no statistically significant difference among the three groups regarding perioperative complications such as hypotension, hallucinations, or nightmares, while there was a statistically significant increase in the incidence of bradycardia in the dexmedetomidine group compared to the control and ketofol groups, with no statistically significant difference detected between the last two groups (Table 2).

**Table 2:** Total fentanyl consumption and complications among the studied groups

Variables	Group C (*n* = 40)	Group D (*n* = 40)	Group K (*n* = 40)	***P***	LSD
**Fentanyl: (μg/kg)**	4.5 ± 0.9	3.3 ± 0.8	3.5 ± 0.8	^†^ < 0.001**	** < 0.05**^1^
					** < 0.05**^2^
					> 0.05^3^
**Emergence agitation N (%):**					** < 0.05**^1^
**No:**	19 (47.5%)	30 (75%)	27 (67.5%)	^‡^0.03*	> 0.05^2^
**Yes:**	21 (52.5%)	10 (25%)	13 (32.5%)		> 0.05^3^
**Hypotension N (%):**					> 0.05^1^
**No:**	37 (92.5%)	34 (85%)	37 (92.5%)	^‡^0.435	> 0.05^2^
**Yes:**	3 (7.5%)	6 (15%)	3 (7.5%)		> 0.05^3^
**Bradycardia N (%):**					** < 0.05**^1^
**No:**	40 (100%)	31 (77.5%)	40 (100%)	^‡^ < 0.001**	> 0.05 ^2^
**Yes:**	0 (0%)	9 (22.5%)	0 (0%)		** < 0.05**^3^
**Hallucination N (%)**					> 0.05^1^
**No:**	39 (97.5%)	40 (100%)	36 (90%)	^‡^0.06	> 0.05^2^
**Yes:**	1 (2.5%)	0 (0%)	4 (10%)		> 0.05^3^
**Nightmares N (%):**					> 0.05^1^
**No:**	40 (100%)	40 (100%)	40 (100%)		> 0.05^2^
**Yes:**	0 (0%)	0 (0%)	0 (0%)	^‡^1.00	> 0.05^3^

Data are expressed as the mean ± SD, number and percentage

*C* Control group, *D* Dexmedetomidine group, *K* Ketofol group

*n* = Total number of patients in each group

^†^ANOVA test

^‡^Chi-square test, LSD (least significance difference)

Non significant difference (*P* > 0.05)

^*^Significant difference (*p* < 0.05)

^**^highly significant difference (*p* < 0.001)

^1^Control group versus dexmedetomidine group

^2^Control group versus ketofol group

^3^ketofol group versus dexmedetomidine group

The incidence of POD assessed by the CAM-ICU score was statistically significantly higher in the control group than in the dexmedetomidine and ketofol groups at all postoperative time points (with no statistically significant difference between the two interventional groups) (Table 3).

**Table 3:** Postoperative delirium at different postoperative times assessed by the CAM-ICU score among the studied groups

Variables	Group C (*n* = 40)	Group D (*n* = 40)	Group K (*n* = 40)	***P***	Chi for trend
**2 h postop:**					** < 0.05**^**1**^
**No:**	31 (77.5%)	38 (95%)	38 (95%)	0.01*	** < 0.05**^**2**^
**Yes:**	9 (22.5%)	2 (5%)	2 (5%)		> 0.05^3^
**1st day evening:**					< **0.05**^**1**^
**No:**	32 (80%)	39 (97.5%)	38 (95%)	0.03*	** < 0.05**^**2**^
**Yes:**	8 (20%)	1 (2.5%)	2 (5%)		> 0.05^3^
**2nd day at 11 a.m.:**					** < 0.05**^**1**^
**No:**	33 (82.5%)	39 (97.5%)	38 (95%)	0.03*	** < 0.05**^**2**^
**Yes:**	7 (17.5%)	1 (2.5%)	2 (5%)		> 0.05^3^
**2nd day at 6 p.m.:**					** < 0.05**^**1**^
**No:**	34 (85%)	39 (97.5%)	39 (97.5%)	0.03*	** < 0.05**^**2**^
**Yes:**	6 (15%)	1 (2.5%)	1 (2.5%)		> 0.05^3^
**3rd day at 11 a.m.:**					** < 0.05**^**1**^
**No:**	34 (85%)	40 (100%)	39 (97.5%)	0.03*	** < 0.05**^**2**^
**Yes:**	6 (15%)	0 (0%)	1 (2.5%)		> 0.05^3^
**3rd day at 6 p.m.:**					** < 0.05**^**1**^
**No:**	36 (90%)	40 (100%)	40 (100%)	0.01*	** < 0.05**^**2**^
**Yes:**	4 (10%)	0 (0%)	0 (0%)		> 0.05^3^

Data are expressed as numbers and percentages

*C* Control group, *D* Dexmedetomidine group, *K* Ketofol group, *CAM-ICU* Confusion Assessment Method for ICU

*n* = Total number of patients in each group. Chi-square test

Non significant difference (*P* > 0.05)

^*^Significant difference (*p* < 0.05)

^1^Control group versus dexmedetomidine group

^2^Control group versus ketofol group

^3^ketofol group versus dexmedetomidine group

There was statistically significant relation between the incidence of POD and the emergence agitation and fentanyl consumption (Table 4, 5), where the higher incidence of POD occurred among those patients developed emergence agitation during recovery and those consumed higher doses of rescue fentanyl in the postoperative period.

**Table 4:** Relationship between the occurrence of emergence agitation and POD assessed by the CAM-ICU score at different times among the studied groups

Variables	Patients without emergence agitation (*n* = 76)	Patients with emergence agitation (*n* = 44)	*P* value
POD 2 h postop:			
No:	72 (94.7%)	35 (79.5%)	0.009*
Yes:	4 (5.3%)	9 (20.5%)	
POD 1st day evening:			
No:	72 (94.7%)	37 (84.1%)	0.04*
Yes:	4 (5.3%)	7 (15.9%)	
POD 2nd day at 11 a.m.:			
No:	73 (96.1%)	37 (84.1%)	0.02*
Yes:	3 (3.9%)	7 (15.9%)	
POD 2nd day at 6 p.m.:			
No:	73 (96.1%)	39 (88.6%)	0.116
Yes:	3 (3.9%)	5 (11.4%)	
POD 3rd day at 11 a.m.:			
No:	72 (94.7%)	41 (93.2%)	0.72
Yes:	4 (5.3%)	3 (6.8%)	
POD 3rd day at 6 p.m.:			
No:	76 (100%)	40 (90.9%)	0.007*
Yes:	0 (0%)	4 (9.1%)	

Data are expressed as numbers and percentages

*POD* Postoperative delirium, *CAM-ICU* Confusion Assessment Method for ICU

*n* = Total number of patients in each group. Chi-square test

Non significant difference (*P* > 0.05)

^*^Significant difference (*p* < 0.05)

**Table 5:** Relationship between occurrence of POD and cumulative fentanyl dose among the studied groups

Variables	Fentanyl dose Mean ± SD	*P* value
**POD 2 h postop:**		
**No:**	3.74 ± 1.03	0.01*
**Yes:**	4.46 ± 1.05	
**POD 1st day evening:**		
**No:**	3.77 ± 1.05	0.130
**Yes:**	4.30 ± 0.94	
**POD 2nd day at 11 a.m.:**		
**No:**	3.75 ± 1.02	0.01*
**Yes:**	4.67 ± 1.11	
**POD 2nd day at 6 p.m.:**		
**No:**	3.80 ± 1.06	0.392
**Yes:**	4.33 ± 0.57	
**POD 3rd day at 11 a.m.:**		
**No:**	3.81 ± 1.05	0.805
**Yes:**	4 ± 1.41	
**POD 3rd day at 6 p.m.:**		
**No:**	3.80 ± 1.04	0.405
**Yes: **	4.25 ± 1.5	

Data are expressed as the mean ± SD

*POD* Postoperative delirium *n* = Total number of patients in each group

Independent sample t test

Non significant difference (*P* > 0.05)

^*^Significant difference (*p* < 0.05)

There was a significant positive correlation between the fentanyl dose received by the patient and the occurrence of POD 2 h postoperatively (*r* = 0.227, *p* = 0.01) and on the morning of the second postoperative day (*r* = 0.239, *p* = 0.009) (Table 6).

**Table 6:** The correlation between fentanyl dose and occurrence of POD at different times among the studied group

Variable	Fentanyl dose	
	**R**	***p***
**POD 2 h postop**	**0.227**	0.01*
**POD 1st day evening:**	0.166	0.070
**POD 2nd day at 11 a.m**	**0.239**	0.009*
**POD 2nd day at 6 p.m**	0.095	0.3041
**POD 3rd day at 11 a.m**	0.022	0.815
**POD 3rd day at 6 p.m**	0.093	0.310

*POD* Postoperative delirium

r: correlation coefficient

Non significant difference (*P* > 0.05)

^*^Significant difference (*p* < 0.05)

The logistic regression model revealed that the occurrence of emergence agitation was a significant dependent predictor for the occurrence of postoperative delirium measured by the CAM-ICU score at 2 h postoperatively (odds ratio [OR], 4.35; CI, 1.46 to 12.9) and on the evening of the 1st postoperative day (OR, 3.69; CI, 1.14 to 11.9). Furthermore, a high dose of fentanyl intake was found to be another significant dependent predictor at 2 h postoperatively (OR, 2.13; CI, 1.23 to 3.68) as well (Table 7).

**Table 7:** Logistic regression analysis of factors predicting POD at different times among the studied groups

Independent factors	B	S.E	Wald	O.R (95% C.I)	*P* value
**POD 2 h postop:**					
Emergence agitation	1.470	0.556	6.995	4.35(1.46–12.9)	0.008*
Fentanyl dose	0.759	0.278	7.447	2.13 (1.23–3.68)	0.006*
**POD 1st day evening:**					
Emergence agitation	1.307	0.599	4.763	3.69(1.14–11.9)	0.02*
Fentanyl dose	0.269	0.279	0.932	1.30(0.75–2.26)	0.334
**POD 2nd day at 11 a.m**					
Emergence agitation	1.072	0.611	3.079	2.92(0.88–9.68)	0.079
Fentanyl dose	0.554	0.295	3.410	1.72(0.96–3.06)	0.065
**POD 3rd day at 6 p.m**					
Emergence agitation	1.931	1.155	2.793	6.89(0.71–66.04)	0.095
Fentanyl dose	0.172	0.457	0.141	1.18(0.48–2.91)	0.707

*POD* Postoperative delirium

*B* Unstandardized coefficient, *SE* Standard error, *Wald* Wald chi-square test, *OR* Odds ratio, *CI* Confidence interval

Non significant difference (*P* > 0.05)

^*^Significant difference (*p* < 0.05)

## Discussion

Postoperative delirium is a significant complication in elderly patients that has been reported as a clinical challenge for the anesthetists in the recovery process of these patients, so preventing and treating POD has received widespread attention. Many studies have evaluated the efficacy of perioperative drug administration for POD prevention such as ketamine, propofol and DEX [1, 19, 25]. Most of these studies concluded that the mentioned drugs were effective in minimizing POD incidence, but they also focused clearly on DEX as the first choice for this purpose [26]. Our study was designed to evaluate a safe and simple method to decrease the incidence of POD in elderly patients scheduled for urgent exploration due to intestinal obstruction by continuously infusing ketofol (a mixture of ketamine and propofol at a ratio of 1:4) intraoperatively and for 2 h postoperatively compared to DEX.

Ketofol and DEX were favored in our study as both produce pain relief and sedation. DEX, a selective alpha 2 agonist with sedative, anxiolytic, and analgesic effects has been promoted by many studies because of its ability to produce levels of semi-arousable and cooperative sedation without risk of respiratory depression [16, 17, 26].

A lower incidence of POD was reported with DEX administration than with propofol administration [3.0% vs 6.6%, respectively] in Shin et al. study on healthy older adults undergoing lower extremity orthopedic surgery [27]. Additionally, Liu et al. declared in their meta-analysis that DEX could reduce POD and was associated with a shorter length of intubation compared to propofol but might increase bradycardia in patients after cardiac surgery [28]. However, Li et al. found that propofol slightly impaired the cognitive function in elderly patients undergoing elective unilateral total hip replacement surgery and it demonstrated a significant advantage in postoperative cognitive dysfunction incidence compared to DEX and midazolam [25]. In another study, Jiguo et al. revealed that DEX and propofol were effective in patients with POD, but DEX was associated with fewer adverse reactions [26]. Again, Hughes et al., in their multicenter trial, showed that mechanically ventilated adults with sepsis who received DEX did not differ in the number of days alive without delirium or coma from those who received propofol [29].

Ketofol is the combination of ketamine and propofol. Low doses of ketamine are well known to produce effective analgesia and opioid-sparing effects [9]. Also, propofol in low doses produces sedation and decreases emergence agitation depending on the administration time [11]. We opted to use ketofol in our study as it combines the properties of both ketamine and propofol to decrease the emergence agitation incidence and severity, guarantees hemodynamic stability with good post-operative analgesia, sedation, and recovery [13–15].

Ketamine infusion in elderly patients undergoing cardiac surgery with cardiopulmonary bypass in Siripoonyothai & Sindhvananda was found to lower the incidence of 24-h POD compared to propofol infusion. They justified their findings as ketamine maintained a higher MAP during CPB, which can lead to higher cerebral blood flow and subsequently higher cerebral oxygenation. Additionally, given that older patients frequently have a history of depression, and that postoperative inflammation severity was found to be a significant predictor of 24-h POD, its antidepressant and anti-inflammatory effects may also be relevant [30]. Moreover, a meta-analysis provided by Hovaguimian et al. clarified that a bolus dose of ketamine at the induction of anesthesia led to a 65% decrease in the risk of postoperative cognitive dysfunction (POCD) in patients undergoing cardiac, abdominal, or orthopedic surgery [31]. Although a previous study demonstrated that ketamine failed to reduce the incidence of POD in patients undergoing major surgery [32], we believe that ketamine is noninferior to DEX in the prevention of POD.

In our randomized study, older patients operated for an emergency intestinal obstruction showed a lower incidence of POD in the ketofol and DEX groups than in the control group at all postoperative time points and regarding the effectiveness of ketofol in decreasing the POD incidence, it was as effective as DEX with no statistically significant difference was observed between the two interventional groups. Moreover, more stable hemodynamics and lower postoperative pain severity were recorded with ketofol administration in this study.

In the current study, the mean values of HR and MAP were statistically significantly lower in DEX group than in the control and ketofol groups and the ketofol group had the least fluctuation in the hemodynamic parameters during all measurement times until the end of drug infusion. This may be attributed to the fact that ketamine and propofol appear to counter each other’s adverse effects, enhancing the advantage of ketofol as a hemodynamic stabilizer, unlike DEX, which was associated with a significant increase in the incidence of bradycardia. In accordance with this result, Zeng et al., in their meta-analysis of DEX administration in elderly patients undergoing noncardiac surgery for prevention of POD, found that DEX was linked to the increased risk of perioperative bradycardia with no significant effect on the occurrence of perioperative hypotension when compared with placebo [33]. On the other hand, Ali et al. reported satisfactory hemodynamic stability and breathing ratio with a blend of 0.15 mg/kg ketamine and 0.45 mg/kg propofol (1:3) in comparison to the ketofol untreated group in children undergoing adenotonsillectomy [14], and Yalcin et al. clarified that ketofol (1:1) had better hemodynamic stability, without any important side effects, than ketamine and propofol groups in electroconvulsive therapy anesthesia [34]. Maheswari et al. declared that ketofol for the induction and maintenance of anesthesia during decompressive craniectomy in patients with traumatic brain injury was associated with more hemodynamic stability and lower vasopressor requirements than propofol [35].

Also, lower postoperative pain scores were observed in both DEX and ketofol groups than in the control group in our study with no significant difference found between the DEX and ketofol groups at all postoperative time points except at 48 and 72 h, where the values of the three studied groups were comparable. Consequently, it has been reflected on the total consumed fentanyl in the postoperative three days, as it was significantly higher in the control group compared to the other two interventional groups. These findings were matched with Ali et al. where the numeric rating of postoperative pain presented a significant decrease in postoperative pain in the ketofol group that led to a comfortable awake up and eliminated postsurgical pain therapy in children undergoing adenotonsillectomy [14].

Our results showed a significant increase in the incidence of emergence agitation in control group compared to DEX and ketofol groups, with no significant difference between DEX group and ketofol group. Similar to these findings, Ali et al. found that ketofol at a dose of ketamine 0.25 mg/kg in combination with propofol 1 mg/kg was as effective as DEX at a dose of 0.3 μg/kg for the prevention of emergence delirium in children undergoing orthopedic surgery with sevoflurane-based anesthesia but with a better analgesic effect and without delaying emergence [36]. Another study by Jayaraj et al. revealed that DEX was more effective than ketofol in reducing emergence agitation in children undergoing adenotonsillectomy (20% vs 28%, respectively, at T0 = when the child had first response to command). Over time, at 20 and 30 min later, none of the patients developed emergence agitation in either group, but there was prolongation of extubation time and time of discharge from PACU in DEX group. The severity of emergence delirium was comparable in both groups [37].

Ali et al. showed that the incidence and severity of emergence agitation were significantly lower in ketofol group than in control group, with percentages of 13.33% vs 48.33% and 8% vs 15%, respectively (*P* < 0.05) [14]. Additionally, DEX and ketamine, according to Chen et al. prevent postoperative emergence agitation following sevoflurane anesthesia for pediatric strabismus surgery, as the incidence of emergence agitation was lower in the dexmedetomidine and ketamine groups (*P* < 0.001, *P* = 0.002, respectively) than in the placebo group [38].

From the previous studies, although DEX could currently be recommended as the first choice for decreasing the incidence of POD, ketamine and propofol could also have a comparable effect regarding POD incidence according to the aforementioned studies. Although, the research using ketofol administration (a mixture of ketamine and propofol) to decrease POD incidence and severity is scarce, the authors believed that it would have a similar effect to its components and our results confirmed that ketofol (a mixture of ketamine and propofol at a ratio of 1:4) was as effective as DEX at a dose of 0.2 µg/kg/h in lowering POD incidence with more stable hemodynamics and lower postoperative pain severity with ketofol administration. Furthermore, ketofol is considered more economical than DEX, which may give it a special advantage in developing countries.

In another aspect of our study, it was found that the occurrence of emergence agitation and high-dose fentanyl consumption postoperatively were significant predictors of the occurrence of postoperative delirium at 2 h and on the evening of the 1st postoperative day. In support of these findings, Zhang et al. in their observational study [39], and other studies [40–42] reported that emergence delirium is independently associated with an increased risk of POD in elderly patients admitted to PACU after major surgery and general anesthesia. Jin et al. also mentioned in their research [43] that many observational studies have shown that a higher postoperative pain score and the use of opioids have been associated with an increased risk of POD [44–46].

Therefore, these results recommend and entrench the idea that taking preventive measures to decrease the incidence of emergence delirium via different anesthetic techniques and proper management of postoperative pain via different multimodal approaches, thus subsequently reflecting on decreasing postoperative opioid consumption, could be an important and effective requirement to reduce the incidence of POD. It is worth noting that, fortunately, these study drugs have a long and influential history in dealing with the abovementioned obstacles.

To the best of the authors’ knowledge, this is the first study to discuss the use of ketofol for the prevention of POD in elderly patients with such a regimen of infusion, the enrollment of cases before surgery with an extensive baseline evaluation and preparation to decrease risk factors, and the continuous rigorous collection of comprehensive data on the daily progress of each elderly patient using CAM score to assess POD and linking the results to detect the possible risk factors that could increase the incidence of its occurrence.

However, there were some limitations. First, the POD rates reported may have been underestimated, as more vulnerable subjects with neuropsychiatric disturbances were excluded in this study. Second, this was a single-center study involving emergency elderly patients who tend to be more compromised than elective ones, which might result in bias while analyzing the data. Third, intraoperative infusion of ketofol in a mixed ratio of 1:4 only was used, although it has been used for different purposes in different mixed ratios and timings. Therefore, further studies on large-scale subjects, different patient subgroups and use of different mixed ratios are required to elucidate who would benefit more and to recommend the most effective ratio with the least drawbacks to be used.

## Conclusion

In conclusion, the administration of ketofol (a mixture of ketamine and propofol at a ratio of 1:4) provides a promising alternative option that is as effective as DEX in reducing the incidence of postoperative delirium and pain in elderly patients scheduled for urgent exploration for intestinal obstruction. Additionally, emergence agitation and high-dose fentanyl consumption on the 1st postoperative day were found to be significant predictors for the occurrence of POD.

### Supplementary Information

**Additional file 1.** Reporting checklist for randomised trial.

## Data Availability

The data used and analyzed during our study are available from the corresponding author on reasonable request.

## Abbreviations

CAM ICU: Confusion assessment method for the ICU
CVP: Central venous pressure
DEX: Dexmedetomidine
ETCO_2_: End tidal Carbon dioxyde.
GABA: γ-Aminobutyric acid receptor
MAP: Mean arterial pressure.
PACU: Postoperative care unit
POD: Postoperative delirium
POCD: Postoperative cognitive dysfunction
RASS: Richmond agitation and sedation score
SBO: Small bowel obstruction
SPMSQ: Short Portable Mental Status Questionnaire
UOP: Urine output
VAS: Visual analogue scale
